# The Metric System

**Published:** 1880-08

**Authors:** 


Of the Metric System, which has been formally adopted by the American Medical Association, the North Carolina Medical Journal very pertinently remarks:

We need reforms of far greater importance than changing weights and measures, and it is not worth while to be so far in advance of public action as to expect no following at all, especially as the movement does not involve any matters of vital interest.—*American Practitioner.*

